# Fructan metabolism and changes in fructan composition during cold acclimation in perennial ryegrass

**DOI:** 10.3389/fpls.2015.00329

**Published:** 2015-05-12

**Authors:** Shamila W. Abeynayake, Thomas P. Etzerodt, Kristina Jonavičienė, Stephen Byrne, Torben Asp, Birte Boelt

**Affiliations:** ^1^Department of Agroecology, Aarhus UniversitySlagelse, Denmark; ^2^Department of Molecular Biology and Genetics, Aarhus UniversitySlagelse, Denmark; ^3^Laboratory of Genetics and Physiology, Institute of Agriculture, Lithuanian Research Centre for Agriculture and ForestryAkademija, Lithuania

**Keywords:** cold acclimation, degree of polymerization, fructan biosynthesis, fructan metabolism, gene, ryegrass

## Abstract

Perennial ryegrass (*Lolium perenne* L.) produces high levels of fructans as a mixture of oligosaccharides and polysaccharides with different degrees of polymerization (DP). The present study describes the analysis of the compositional changes in the full spectrum of fructans, fructan distribution between above ground biomass (top) and the roots, and the transcription of candidate genes involved in fructan metabolism during cold acclimation in perennial ryegrass variety “Veyo” and ecotype “Falster” from distinct geographical origins. We observed changes in fructan composition and induction of low-DP fructans, especially *DP* = 4, in both the top and the roots of “Veyo” and “Falster” in response to low-temperature stress. The accumulation of *DP* > 50 fructans was only apparent in the top tissues where the *Lp1-FFT* expression is higher compared to the roots in both “Veyo” and “Falster.” Our results also show the accumulation and depolymerization of fructans with different DP, together with the induction of genes encoding fructosyltransferases and fructan exohydrolases in both “Veyo” and “Falster” during cold acclimation, supporting the hypothesis that fructan synthesis and depolymerization occurring simultaneously. The ecotype “Falster,” adapted to cold climates, increased total fructan content and produced more *DP* > 7 fructans in the roots than the variety “Veyo,” adapted to warmer climates. This indicates that high-DP fructan accumulation in roots may be an adaptive trait for plant recovery after abiotic stresses.

## Introduction

Changes in the content and composition of water-soluble carbohydrates in response to abiotic stresses are important metabolic re-adjustments in temperate grasses (Rao et al., 2011; Abdelgawad et al., 2014). The water-soluble polymeric sugars, fructans are major reserve carbohydrates in temperate grasses. A number of mechanisms have previously been suggested to explain the role of fructans in abiotic stress tolerance. These include the stabilization of cell membranes to reduce water leakage (Hincha et al., 2000), the prevention of cell volume reduction by increasing osmotic pressure, and freezing point depression (Krasensky and Jonak, 2012). Fructans also play a role as antioxidants that scavenge reactive oxygen species (ROS) and prevent cellular damage during abiotic stress conditions (Bolouri-Moghaddam et al., 2010; Peshev et al., 2013). The likely scenario is that fructans improve stress tolerance via a combination of several mechanisms.

Plant fructans are diverse in molecular structure and weight, degree of polymerization (DP), and linkage types between fructosyl residues. Five major types of fructans are known: inulin, levan, inulin neoseries, levan neoseries, and branched graminan-type fructans (Vijn and Smeekens, 1999; Van den Ende, 2013). Perennial ryegrass (*Lolium perenne* L.) contains the inulin series, inulin neoseries, and levan neoseries-type fructans with both β-(2→1)-linked and β-(2→6)-linked fructosyl residues (Rasmussen et al., 2013). The structural diversity of fructans is mainly controlled by fructosyltransferases (FTs). A number of FTs involved in fructan biosynthesis, such as sucrose:sucrose 1-fructosyltransferase (1-SST), fructan:fructan 1-fructosyltransferase (1-FFT), sucrose:fructan 6-fructosyltransferase, and fructan:fructan 6G-fructosyltransferase (6G-FFT), have been characterized from different plant species (Van den Ende et al., 1996; Lasseur et al., 2006, 2011). Fructan exohydrolases (FEHs) are involved in the degradation of fructans by releasing terminal fructosyl residues. FTs and FEHs belong to the family of glycoside hydrolases and share high amino acid sequence similarity. Other members of the same gene family, such as vacuolar invertases, may also produce small fructans (kestoses), when challenged with high sucrose (De Coninck et al., 2005; Lasseur et al., 2009).

A period of low temperature stress (cold acclimation) induces morphological, physiological, and biochemical changes in both shoot and root tissues that are required for the acquisition of freezing tolerance in cold-tolerant plants (Kerr and Carter, 1990; Goulas et al., 2003; Hoffmann et al., 2010). The alterations in cytoskeletal structures (Kerr and Carter, 1990), plasma membrane lipid alterations (Sassaki et al., 2013), accumulation of compatible solutes (Castonguay et al., 1995) has been shown in the root tissues. Cold acclimation has been shown by upregulation of proteins involved in stress responses and metabolic activities such as glycolysis, nucleoside metabolism, and carbohydrate metabolism. Studies have shown an alteration of fructan metabolism during low-temperature stress. Carbon storage in roots in response to cold exposure has been demonstrated in plants adapted to temperate regions (Prud'homme et al., 1993; Puebla et al., 1997; Goulas et al., 2003) suggesting resource allocation toward storage organs as a strategy for plant recovery after freezing. Fructan content in perennial grass roots varied over the year with a minimum in early spring, when fast recovery is necessary (Steen and Larsson, 1986). De Roover et al. (1999) also demonstrated utilization of fructan reserve by rapid induction of 1-FEH in chicory roots during plant recovery after defoliation. Analysis of total fructan content alone, during the development of freezing tolerance does not provide information about the changes in fructan composition in response to low-temperature stresses and further there is a lack of knowledge about the effect of the fructan distribution between shoot and root tissues in contrasting ecotypes adapted to different climatic conditions (De Roover et al., 2000; Hisano et al., 2008; Rao et al., 2011). Analysis of the full spectrum of fructans has previously been restricted by a limited availability of methods with the capacity to analyse at a high mass range. Of the numerous methods that have been tried (John et al., 1996; Lopez et al., 2003; Harrison et al., 2011), high resolution time-of-flight mass spectroscopy (TOF-MS), which measures the mass-to-charge ratio of pulled ions, can distinguish fructan polymers from other molecular species with mass-to-charge ratios similar to those of fructans.

The present study focuses on the contents and compositional changes in the full spectrum of fructans and on the gene expression patterns of FTs and FEHs during cold acclimation in perennial ryegrass from distinct geographical origins. The investigations included two types of perennial ryegrass: The ecotype “Falster” which originates from Denmark and is adapted to cold climates, and the variety “Veyo” originating from Italy and adapted to warmer climates (Jensen et al., 2005). The relative quantification of fructans with different DPs was determined using high-resolution liquid chromatography–electrospray ionization TOF-MS (LC-ESI-TOF-MS) and gene expression analysis was performed using quantitative RT-PCR in both “Veyo” and “Falster” during cold acclimation.

## Materials and methods

### Plant materials and growth conditions

Perennial ryegrass (*Lolium perenne* L.) variety “Veyo” originating from Italy and adapted to warmer climates, and ecotype “Falster” originating from Denmark and adapted to cold climates (Jensen et al., 2005) were propagated vegetatively in a glasshouse. Following propagation, the plants were grown in a glasshouse (photoperiod of 9 h:15 h, light:dark) at ~20°C for 12 weeks. The plants (at the pre-elongation stage) were then transferred to a growth chamber for 7 d (photoperiod of 9 h:15 h, light:dark), with 450 μmol photons m^−2^ s^−1^ light intensity at 20°C. For induction of fructan biosynthesis, the plants were cold-acclimated in a controlled growth room for 17 d (photoperiod of 9 h:15 h, light:dark), with 450 μmol photons m^−2^ s^−1^ light intensity and relative humidity of ~70% at 7°C. The chlorophyll fluorescence of leaves was measured, and root and above ground biomass (top) samples were harvested in three biological replicates at the onset of the daily photoperiod on d 0, 1, 3, 5, 9, 13, and 17 of cold acclimation at 7°C. The samples were frozen in liquid nitrogen immediately after harvesting and stored at -80°C, until the analysis of carbohydrates and gene expression levels was performed. After d 17 of cold acclimation, the “Veyo” and “Falster” plants were transferred to a freezing chamber at −10°C and frozen for 3 h. As a control, non-cold-acclimated “Veyo” and “Falster” plants were also transferred to the same freezing chamber and frozen for 3 h. The chlorophyll fluorescence and electrolyte leakage of leaves were measured before freezing, and after 30 and 120 min of freezing.

### Measurement of chlorophyll fluorescence

Chlorophyll fluorescence measurements were made using a pulse amplitude modulated fluorometer (MINI-PAM) (Walz, Effeltrich, Germany). The measurements were taken from three biological replicates, from three mature leaves of each plant (3 cm above the leaf sheath). The quantum yield of photosystem II (Φ_PSII_) was calculated, as previously described (Zhang et al., 2008).

### Measurement of electrolyte leakage

To determine the electrolyte leakage, 0.1 g of fully developed leaves were harvested and washed three times with deionized water. The electrolyte leakage of leaves was measured, as previously described (Hu et al., 2012). The measurements were taken from three biological replicates.

### Measurement of total fructan

Samples were freeze-dried and ground to powder. Soluble carbohydrates were extracted from 100 mg of sample with 25 ml of 0.1 M acetate buffer (pH 5.0) for 1 h at 65°C. Extracts from each sample (2 ml) were hydrolyzed using an equal volume of 0.074 M H_2_SO_4_ for 70 min at 80°C. The amounts of glucose and fructose before and after acid hydrolysis were measured, using the hexokinase–phosphoglucose isomerase glucose-6-phosphate dehydrogenase system, by calculating the sucrose and fructan contents, as previously described (Rao et al., 2011).

### Analysis of fructan composition

Ground plant (100 mg) material was transferred into a 33 ml stainless cell (Dionex, Denmark) for accelerated solvent extraction and mixed with 10 g of Ottawa sand (particle size 20 to 30 mesh, Fischer Chemicals). The top of the cell was then fitted with a cellulose filter (Dionex, Denmark) and filled with glass beads (previously baked at 400°C to remove possible contaminants). Samples were extracted with MilliQ water on the Accelerated Solvent Extractor Dionex ASE 350 system (Unity Lab Services) under the following conditions: one cycle of 5 min pre-heating, 5 min heating at 80°C, 3 min static time, 60 s purge, and 60% flush. Extracts were collected in 52 ml glass cells and weighed to determine the exact extract volume (from density ρ = 0.998 g ml^−1^ at 25°C). Sample extracts (5 ml) were filtered, using Omnifix®-F syringes (B. Braun, Germany) and Q-Max® RR syringe filters (0.45 μm pore size, Frisenette ApS, Denmark), and then lyophilized to dryness and redissolved in 500 μl of MilliQ water, yielding samples 10 times more concentrated for LC-ESI-TOF-MS (Agilent Technologies) analysis.

A total volume of 50 μl of concentrated samples were injected, and fructans were chromatographed on a 100 × 2.1 mm Hybercarb column (5 μm particle size, 250 Å pore size) at 40°C using a flow rate of 0.3 ml min^−1^. Gradient elution from the Hypercarb column was performed, with water and acetonitrile as eluents A and B, respectively, using the following gradient: 15% B at 0–5 min, 15–30% B in 5–30 min, 30–90% B in 30–32 min, 90–2% B in 32–33 min, keep at 2% B at 33–38 min, 2–15% B in 38–39 min, and equilibration at 15% B for 6 min. Ionization of analytes was performed by electrospray ionization in negative polarity, with drying gas temperature of 325°C, dry gas flow rate of 10 l min^−1^, nebulizer pressure of 20.6 × 10^4^ Pa, capillary voltage of 4000 V, and fragmentor voltage of 300 V. Mass spectra were recorded in 4 GHz high-resolution mode on an Agilent 6224 TOF detector in the range of 150–3200 mass-to-charge ratio (*m/z*) and an acquisition rate of 0.66 spectrum s^−1^, yielding the maximum number of transients for TOF detection. Mass accuracy was maintained by the simultaneous infusion of a reference solution of analytical standard compounds through a dual ESI nebulizer. The reproducibility of peak intensities was validated by analysing a reference plant material for all sample batches.

Acquired data were processed in MassHunter Qualitative Analysis software version B.05.00 (Agilent Technologies) extracting ion chromatograms for DP3 to DP70 using a personalized database, including exact masses and chromatographic retention times (Supplementary Table S1). Due to the lack of commercially available analytical standards for fructans, absolute quantification was not possible. Instead, the relative amounts for the same DP were compared for each sampling. Extracting ion chromatograms for each DP contained multiple peaks corresponding to structural isomers, and the area of the major peak was chosen for peak integration.

### RNA extraction and cDNA synthesis

Leaf and root tissues were ground separately in liquid nitrogen. Total RNA was extracted using RNeasy® Plant Mini Kit (Qiagen), and On-column DNAse I digestion (Qiagen) was performed to avoid genomic DNA contamination, according to the manufacturer's instructions. Total RNA quality was verified using an Agilent 2100 Bioanalyzer (RNA 6000 Nano Assay) and quantified by Quant-iT™ RiboGreen® RNA Reagent assay, according to the manufacturer's protocol. First-strand cDNA synthesis was performed using SuperScript II Reverse Transcriptase kit (Invitrogen), as per manufacturer's instructions.

### Quantitative RT-PCR analysis

The quantitative reverse transcriptase-polymerase chain reaction (RT-PCR) standard curve method for absolute quantification was carried out, as previously described (Abeynayake et al., 2012). All reactions were performed in duplicate 10 μl volumes, using SYBR Green Master mix (Applied Biosystems) on an Applied Biosystems® ViiA™ 7 real-time PCR system, according to the manufacturer's instructions. Full-length *L. perenne* cDNA sequences with sequence similarity to fructan-related genes and internal control genes were identified for primer design, querying the *L. perenne* genomic database (Byrne et al., unpublished) with the *Lp1-SST* (AY245431.1) (Chalmers et al., 2003), *Lp1-FFT* (AB186920.1) (Hisano et al., 2008), *Lp6G-FFT* (AF492836.2) (Lasseur et al., 2006), *Lp1-FEH* (DQ016297.2) (Lothier et al., 2007), *Lp6-FEH* (EU219846.1), *Oryza sativa* elongation factor 1-alpha (*OsEF1a*) (D63583.1), *Zea mays* actin (*ZmACT11*) (AY107106.2), and eukaryotic initiation factor 4A (*ZmeIF4a*) sequences. PCR primers (Supplementary Table S2) were designed to amplify the fragment of the gene of interest and cloned in a pCR®2.1-TOPO 3.9 kb vector, according to the manufacturer's instructions (Invitrogen), to use as the DNA standards. Quantitative RT-PCR primers (Supplementary Table S3) were designed within the region amplified for the standard curve. Each quantitative RT-PCR reaction contained 5 μl of SYBR green master mix, optimized concentrations of forward and reverse quantitative RT-PCR primers (200–600 nM), 1 μl of diluted cDNA template in MilliQ water (dilution of 1:4, v:v), and MilliQ water up to 10 μl. Expression values were normalized by geometric averaging of *LpEF1a*, *LpACT11*, and *LpeIF4a* internal control genes.

### Statistical analysis

Statistically significant differences in fructan content, fructan composition, chlorophyll fluorescence, and electrolyte leakage between the two types of ryegrass, distribution between top and root over the 17 d period of cold acclimation were analyzed using the PROC GLM within the Statistical Analysis System Version 9 software package (SAS, Cary, NC, USA). In all cases, the level of significance was set at *P* < 0.05.

## Results

### Fructan accumulation and distribution between top and root tissues

**Figure 1:**
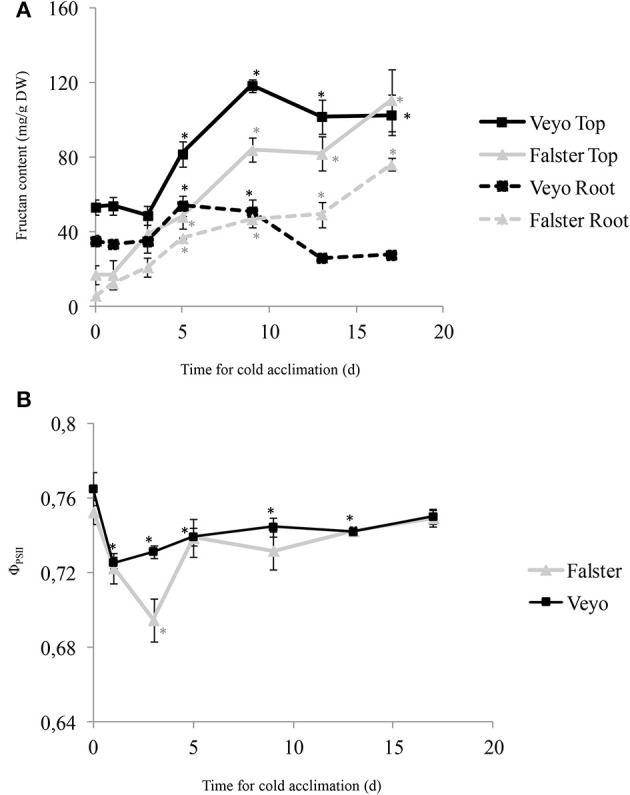
**Fructan accumulation in the green tissues (top) and roots, and the changes in quantum yield of photosystem II (Φ_PSII_) of leaves in perennial ryegrass (*Lolium perenne* L.) variety “Veyo” and ecotype “Falster” during cold acclimation. (A)** Changes in fructan content (mg/g DW) in top and roots of “Veyo” and “Falster.” **(B)** Changes in Φ_PSII_ in the leaves of “Veyo” and “Falster.” Plants were at 20°C on d 0 and at 7°C on d 1–17. Data represent the mean ± SE, obtained from three biological replicates of the analysis. The experiments were repeated twice. Asterisks indicate significant differences (*P* < 0.05) from d 0.

Fructan accumulation in the top and roots of “Veyo” and “Falster” was induced by cold, however, accumulation over time and the distribution between top and root varied in the two types (Figure 1A). In both “Veyo” and “Falster,” a greater content was observed in the top, compared to the roots (*P* < 0.0001). In “Veyo” top, the fructan content increased rapidly from approximately 50 mg/g (d 0–3 of cold acclimation) to 81.8 mg/g at d 5 and it peaked at 118.6 mg/g on d 9. However, in “Veyo” roots, the fructan content increased only slightly to 54 and 50.8 mg/g at d 5 and d 9 and subsequently it decreased to levels similar to the initial level of 35 mg/g. The fructan content increased in the top and roots of “Falster” from 17 and 5.7 mg/g, respectively to 48.1 and 36.6 mg/g on d 5 of cold acclimation, with the highest content recorded on d 17 of cold acclimation. Here, the fructan level increased approximately six-fold in “Falster” top and thirteen-fold in “Falster” roots, compared to the levels on d 0 of cold acclimation.

### Stress levels and survival of plants

The stress levels of the plants during cold acclimation were examined by measuring quantum yield of photosystem II (Φ_PSII_) fluorescence parameter, which measures the efficiency of photosystem II (PSII) photochemistry, indicating the efficiency of carbon fixation (Maxwell and Johnson, 2000), in the leaves. “Veyo” and “Falster” showed a sudden reduction in Φ_PSII_ of leaves when the plants were transferred from 20 to 7°C (Figure 1B). The Φ_PSII_ dropped from d 0 to d 1 in “Veyo,” and from d 0 to d 3 in “Falster.” In “Veyo,” Φ_PSII_ remained beyond the initial level for the whole treatment period, whereas from d 5, Φ_PSII_ was similar to the initial level in “Falster.”

**Figure 2:**
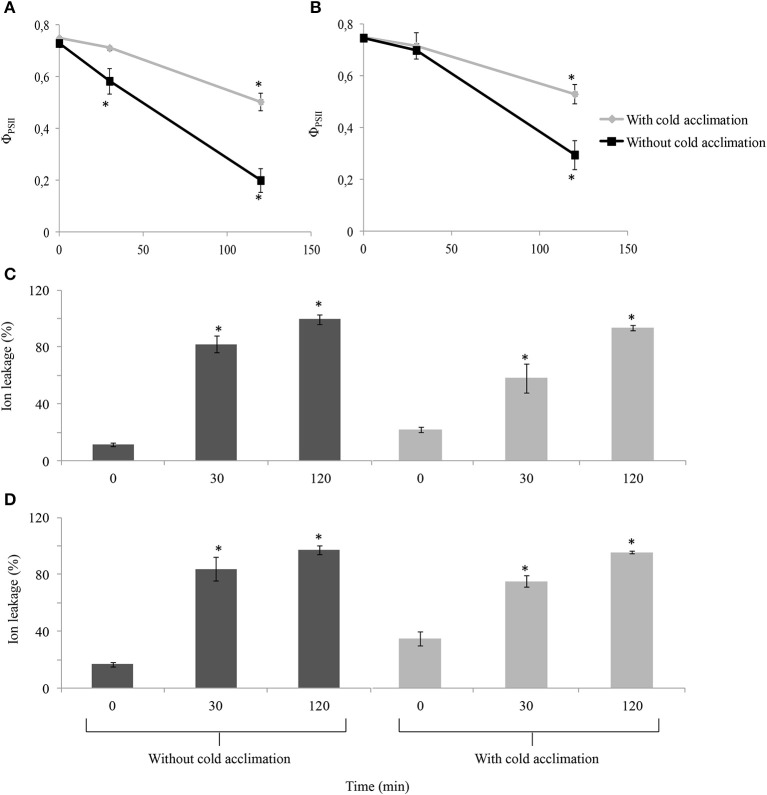
**Changes in quantum yield of photosystem II (Φ_PSII_) and electrolyte leakage of leaves of perennial ryegrass (*Lolium perenne* L.) variety “Veyo” and ecotype “Falster” during freezing at −10°C**. Changes in Φ_PSII_ in **(A)** leaves of “Veyo” and **(B)** leaves of “Falster.” Changes in electrolyte leakage (%) in **(C)** leaves of “Veyo” and **(D)** leaves of “Falster.” Data represent the mean ± SE, obtained from three biological replicates of the analysis. The experiments were repeated twice. Asterisks indicate significant differences (*P* < 0.05) from 0 d in **(A,B)** and from 0 min in **(C,D)**.

After d 17 of cold acclimation, “Veyo” and “Falster” plants were transferred to −10°C for freezing. As a control, non-cold-acclimated plants were also transferred to −10°C for freezing. The stress levels of these plants during freezing were examined by measuring Φ_PSII_ and electrolyte leakage of leaves. Over time the cold acclimated plants were differently affected by freezing than the non-cold-acclimated plants (*P* < 0.0001) and it appeared that “Veyo” and “Falster” responded differently (*P* = 0.07) to the freezing treatment after cold acclimation. In both “Veyo” and “Falster,” a greater reduction of Φ_PSII_ was observed in the non-cold-acclimated, compared to cold-acclimated plants 120 min after the transfer to freezing (Figures 2A,B). The Φ_PSII_ was significantly decreased already after 30 min in the non-cold-acclimated “Veyo” plants whereas “Falster” plants were still unaffected.

Prior to freezing, cold-acclimated plants of both “Veyo” and “Falster” showed a higher electrolyte leakage, compared to the control plants, but, during freezing, they showed a gradual increase in electrolyte leakage (Figures 2C,D). This was in contrast to non-cold-acclimated plants which showed a sudden increase in electrolyte leakage which reached over 80% after 30 min of freezing (Figures 2C,D).

**Figure 3:**
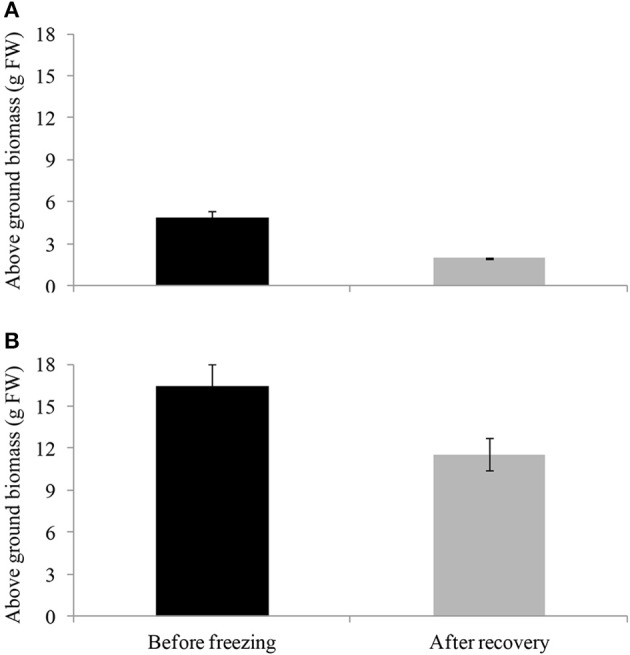
**Recovery of cold-acclimated perennial ryegrass (*Lolium perenne* L.) variety “Veyo” and ecotype “Falster” after freezing**. Fresh weight of above ground biomass before freezing and after 7 d of recovery from freezing (−10°C for 3 h) in **(A)** “Veyo” and **(B)** “Falster.” Data represent the mean ± SE, obtained from three biological replicates of the analysis.

After 3 h of freezing at −10°C, cold-acclimated and non-cold-acclimated plants were transferred to the glasshouse (20°C). Only the cold-acclimated plants from “Veyo” and “Falster” showed a re-growth after 7 d of recovery with “Falster” showing a more vigorous re-growth than “Veyo” (Supplementary Figure S1). Before freezing, “Veyo” and “Falster” had an average fresh weight of above ground biomass 4.86 and 16.43 g, respectively (Figure 3). During the recovery, “Veyo” and “Falster” produced an average fresh weight of above ground biomass 1.94 and 11.54 g, respectively, indicating around 40% of recovery in “Veyo” and around 70% of recovery in “Falster.” Plants without cold acclimation could not be recovered after 3 h of freezing at −10°C.

### Changes in fructan composition during cold acclimation in “Veyo” and “Falster”

**Figure 4:**
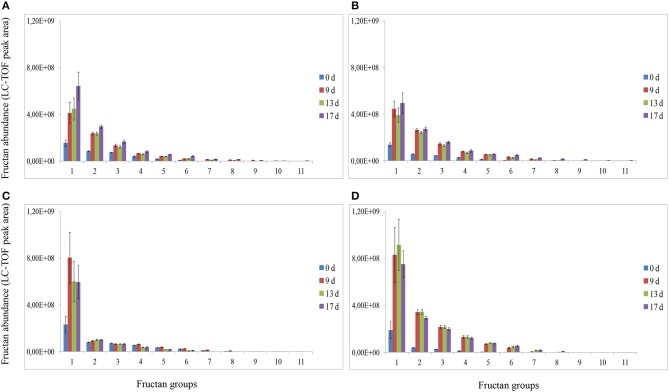
**Changes in fructan DP and composition in perennial ryegrass (*Lolium perenne* L.) variety “Veyo” and ecotype “Falster” during cold acclimation**. Fructan oligosaccharides and polysaccharides were classified into 11 groups, based on their DP, as follows: (1) *DP* = 3–8, (2) *DP* = 9–14, (3) *DP* = 15–20, (4) *DP* = 21–26, (5) *DP* = 27–32, (6) *DP* = 33–38, (7) *DP* = 39–44, (8) *DP* = 45–50, (9) *DP* = 51–56, (10) *DP* = 57–62, and (11) *DP* = 63–70. Changes in fructan DP and composition in the **(A)** green tissues (top) of “Veyo,” **(B)** top of “Falster,” **(C)** roots of “Veyo,” and **(D)** roots of “Falster.” Fructan DP and composition on d 0, 9, 13, and 17 of cold acclimation are shown. Relative quantification of fructan oligosaccharides and polysaccharides was carried out using LC-ESI-TOF-MS. Data represent the mean ± SE, obtained from each group.

**Figure 5:**
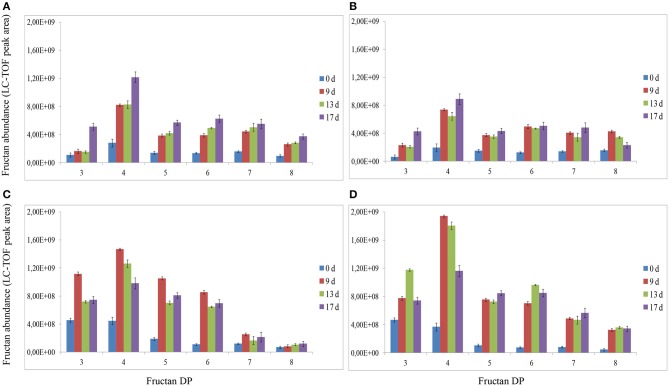
**Changes in fructan DP and composition of low-DP fructans (*DP* = 3–8) in perennial ryegrass (*Lolium perenne* L.) variety “Veyo” and ecotype “Falster” during cold acclimation**. Changes in fructan DP and composition in the **(A)** green tissues (top) of “Veyo,” **(B)** top of “Falster,” **(C)** roots of “Veyo,” and **(D)** roots of “Falster.” Fructan DP and composition on d 0, 9, 13, and 17 of cold acclimation are shown. Relative quantification of fructan oligosaccharides and polysaccharides was carried out using LC-ESI-TOF-MS. Data represent the mean ± SE, obtained from three biological replicates of the analysis.

**Figure 6:**
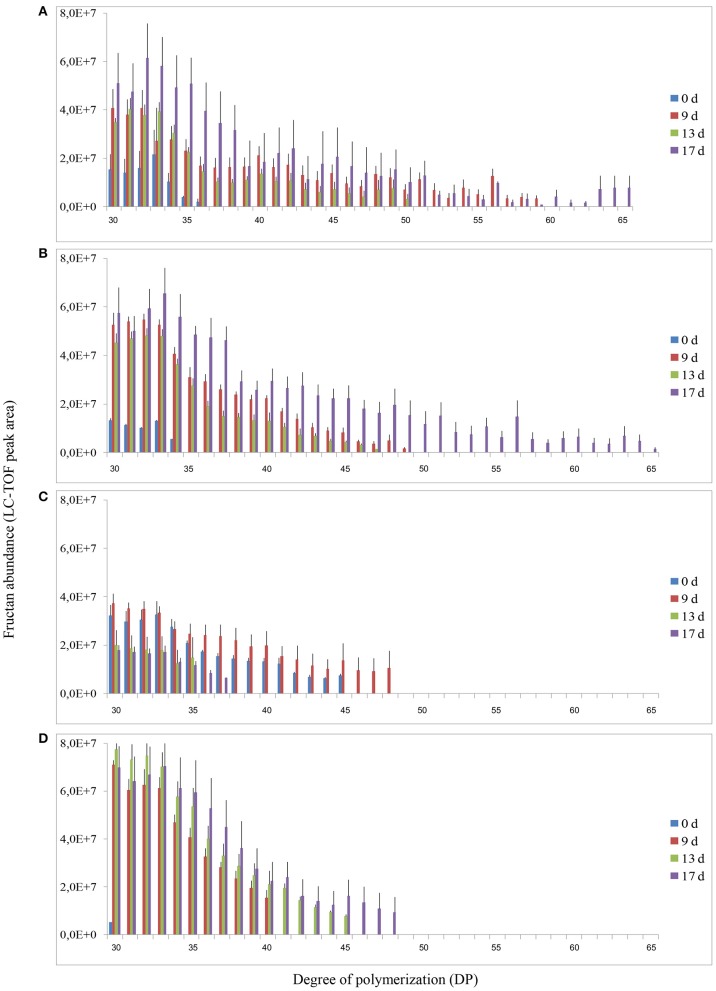
**Changes in fructan DP and composition of high-DP fructans (*DP* > 30) in perennial ryegrass (*Lolium perenne* L.) variety “Veyo” and ecotype “Falster” during cold acclimation**. Changes in fructan DP and composition in the **(A)** green tissues (top) of “Veyo,” **(B)** top of “Falster,” **(C)** roots of “Veyo,” and **(D)** roots of “Falster.” Fructan DP and composition on d 0, 9, 13, and 17 of cold acclimation are shown. Relative quantification of fructan oligosaccharides and polysaccharides was carried out using LC-ESI-TOF MS. Data represent mean ± SE obtained from three replicates of the analysis.

Analysis of fructans was carried out using high-resolution LC-ESI-TOF-MS in top and root samples collected on d 0, 9, 13, and 17. Fructans accumulated as a mixture of oligosaccharides and polysaccharides in “Veyo” and “Falster” and fructans up to *DP* = 70 were identified. For clarification, fructans were classified into 11 groups, based on their DP, as follows: (1) *DP* = 3–8, (2) *DP* = 9–14, (3) *DP* = 15–20, (4) *DP* = 21–26, (5) *DP* = 27–32, (6) *DP* = 33–38, (7) *DP* = 39–44, (8) *DP* = 45–50, (9) *DP* = 51–56, (10) *DP* = 57–62, and (11) *DP* = 63–70. The composition of fructan DP changed in both the above-ground biomass and the roots of “Veyo” and “Falster” during cold acclimation (Figure 4). “Falster” produced more fructans (groups 2–8) in roots than “Veyo” during cold acclimation (Figures 4C,D). Fructans, *DP* = 3–8 were significantly increased in the top and roots of “Veyo” and “Falster” during cold acclimation. Among these low-DP fructans, *DP* = 4 fructan was the most prominent. “Veyo” showed a three-fold increase of *DP* = 4 fructan in both the top and roots (Figures 5A,C), whereas “Falster” showed a four-fold increase in the top and a five-fold increase in the roots (Figures 5B,D). The changes in fructan content at each DP are shown in Supplementary Figure S2. The accumulation of fructans, *DP* > 50 was only apparent in the top tissues of “Veyo” and “Falster” (Figure 6). Our results show that high-DP fructans were reduced from d 9 to d 13 but started to accumulate again from d 13 to d 17 in the top of “Veyo” and “Falster” (Figures 6A,B). The fructans, *DP* > 38 only occurred on d 0 and d 9 in “Veyo” roots. Degradation of fructans, *DP* > 30 from d 9 to d 17 was also observed in “Veyo” roots (Figure 6C), whereas in “Falster” roots fructans with increasing DP (up to *DP* = 48) were accumulated over the 17 day period of cold acclimation. Overall, the composition of the fructan mixture of oligosaccharides and polysaccharides changed in the top and roots of both “Veyo” and “Falster” during cold acclimation.

### Induction of genes involved in fructan metabolism during cold acclimation

**Figure 7:**
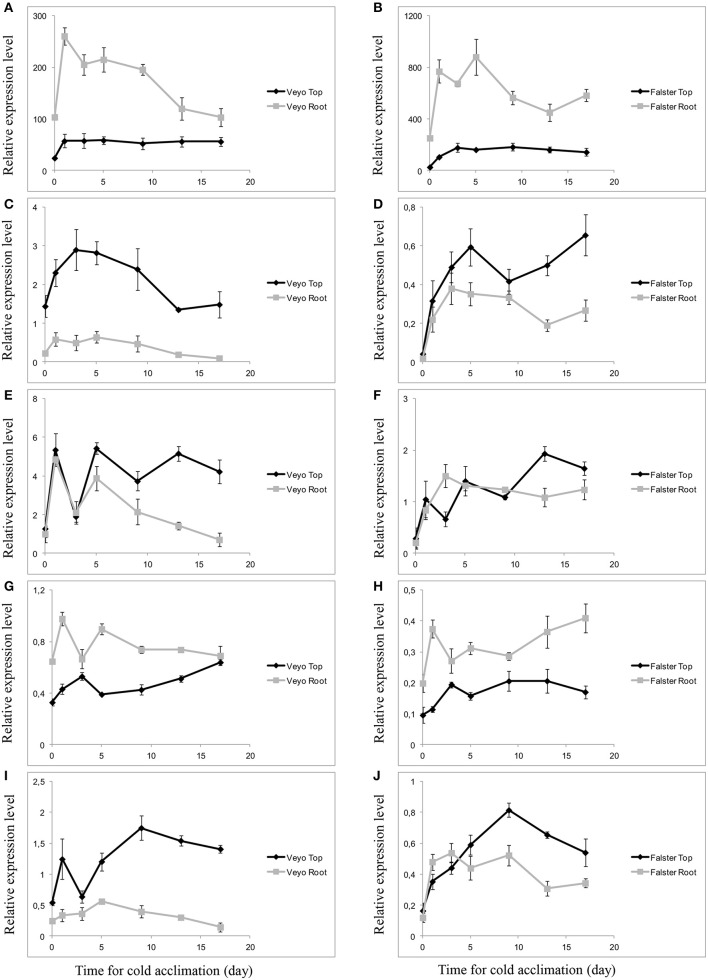
**Differential expression of fructan-related genes in the green tissues (top) and root tissues of perennial ryegrass (*Lolium perenne* L.) variety “Veyo” and ecotype “Falster” during cold acclimation. (A,B)** Sucrose: sucrose 1-fructosyltransferase (*Lp1-SST)*. **(C,D)** Fructan: fructan 1-fructosyltransferase (*Lp1-FFT*). **(E,F)** Fructan: fructan 6G-fructosyltransferase (*Lp6G-FFT*). **(G,H)** Fructan exohydrolase (*Lp1-FEH*). **(I,J)** Fructan exohydrolase (*Lp6-FEH*). Changes in mRNA levels of the genes in the top and root tissues of “Veyo” **(A,C,E,G,I)** and “Falster” **(B,D,F,H,J)** are shown. Relative gene expression was measured by quantitative RT-PCR. Expression values were normalized by geometric averaging of elongation factor 1-alpha (*LpEF1a*), actin (*LpACT11*), and eukaryotic initiation factor 4A (*LpeIF4a*) internal control genes. Data represent the mean ± SE, obtained from three biological replicates of the analysis.

The transcript abundance during cold acclimation was monitored, using quantitative RT-PCR, for the genes *Lp1-SST, Lp1-FFT, Lp6GFFT*, and *Lp1-FEH* with β-(2→1) linkage specificity and *Lp6-FEH* with β-(2→6) linkage specificity. The expression of *Lp1-SST, Lp1-FFT*, and *Lp6G-FFT* was induced after d 1 of cold acclimation in the top and roots of “Veyo” and “Falster.” Expression levels of *Lp1-SST* in the roots were greater than in the top for both “Veyo” and “Falster” (Figures 7A,B). On the other hand, *Lp1-FFT* showed a higher expression in the top, compared to the roots, of both “Veyo” and “Falster” (Figures 7C,D). *Lp6G-FFT* showed similar expression patterns in “Veyo” roots and top up to d 9 of cold acclimation (Figure 7E), after which the expression further decreased in “Veyo” roots. Expression of *Lp6G-FFT* in “Falster” showed an initial peak after d 1 of cold acclimation in the top and after d 3 in the roots (Figure 7F). After d 3 of cold acclimation, *Lp6G-FFT* expression levels in the “Falster” roots remained similar to the initial peak, whilst, in the “Falster” top, a further increase was observed. In “Veyo” and “Falster,” higher expression levels of *Lp1-FEH* were observed in the roots, compared to the top (Figures 7G,H). Moreover, in both “Veyo” and “Falster,” induction of *Lp6-FEH* was observed in the top and roots, with the greatest induction in the top (Figures 7I,J). Much greater induction of *Lp6-FEH* was observed in the “Falster” roots, compared to the “Veyo” roots.

## Discussion

### Physiological responses to cold acclimation in “Veyo” and “Falster”

Numerous studies have previously shown positive correlations between fructan accumulation and cold stress tolerance in plants (Hisano et al., 2004; Li et al., 2007; Kawakami et al., 2008). In addition to the evidence from the studies showing positive correlations between fructan accumulation and cold-stress tolerance, transgenic approaches have also demonstrated the improved freezing tolerance in transgenic plants expressing the genes involved in fructan biosynthesis (Hisano et al., 2004; Li et al., 2007). Our results show an initial drop of fluorescence parameter, Φ_PSII_ in leaves of “Veyo” and “Falster” during cold acclimation. Measurement of Φ_PSII_ has been used to investigate the photosynthetic performance in plants (Oberhuber and Edwards, 1993; Maxwell and Johnson, 2000; Bibi et al., 2008). The quantum yield of photosynthetic electron transport in the PSII protein complex, located in the thylakoid membrane of chloroplast, is related with the efficiency of carbon fixation (Genty et al., 1989). The initial drop of Φ_PSII_ in “Veyo” and “Falster” indicates the adverse effects of increased stress on photosynthetic performance of leaves. Fructan accumulation and increased Φ_PSII_ in leaves were observed after d 3 of cold acclimation in both “Veyo” and “Falster.”

After 17 d of cold acclimation, “Veyo” and “Falster” showed a higher electrolyte leakage in leaves compared to non-cold-acclimated control plants. Increased electrolyte leakage in leaves due to prolonged exposure to low-temperatures has been demonstrated also in other plants (Chang et al., 2001; Campos et al., 2003). This might be due to the chilling injuries in cell membranes, and also due to the increased levels of solutes and cations in cell sap during cold acclimation. The analysis of the stress levels of plants during freezing, by measuring Φ_PSII_ and electrolyte leakage of leaves indicates an improved cold-stress tolerance in cold-acclimated plants of both “Veyo” and “Falster” compared to the non-cold-acclimated plants. The non-cold-acclimated plants also could not be recovered after freezing. An improved cold-stress tolerance in the cold-acclimated plants can be due to induction of fructan metabolism and other protective mechanisms during cold acclimation.

### Changes in fructan composition during cold acclimation

Fructan accumulation and induction of genes involved in fructan metabolism were observed in the top and roots of both “Veyo” and “Falster” during cold acclimation. Differences in fructan contents could be expected between the varieties adapted to different climatic conditions (Rao et al., 2011). However, after 17 d of cold acclimation, total fructan contents in the above ground biomass reached similar levels in both “Veyo” and “Falster,” even though the fructan patterns varied between “Veyo” and “Falster.” The *DP* > 50 fructan fraction increased rapidly in the top tissues, where the Lp1-FFT expression is higher as compared to the roots, in both “Veyo” and “Falster.” 1-FFT is the key enzyme involved in the elongation of fructan chain by adding fructosyl residues from one fructan molecule to another (Lasseur et al., 2006). However, the gene encoding 1-SST involved at the early stage of fructan biosynthesis was highly expressed in roots compared to top in “Veyo” and “Falster.” 1-SST transfers fructosyl residues using sucrose as both donor and acceptor substrates to produce trisaccharide, 1-kestose (Chalmers et al., 2003).

In this study, the concomitant induction of genes encode both FTs and FEHs, and the observation of the reduction of high-DP fructans from d 9 to d 13 but accumulation from d 13 to d 17 in “Veyo” and “Falster” during cold acclimation indicate the simultaneous occurrence of fructan synthesis and degradation. A recent study of carbon fluxes in major carbohydrate pools using ^13^CO_2_ labeling of plants also indicates the occurrence of fructan synthesis and degradation simultaneously in perennial ryegrass (Lattanzi et al., 2012). Co-expression of 1-SST and 1-FEH in wheat stems, has been previously demonstrated, suggesting that FEHs might act as β-(2→1)-trimmers during fructan biosynthesis, and relative proportions and specificities of both FTs and FEHs are important for quantitative and qualitative changes in fructan (Bancal and Triboi, 1993; Van den Ende et al., 2003).

Changes in fructan composition and induction of low-DP fructans, especially *DP* = 4, in both the above ground biomass and the roots of “Veyo” and “Falster” were observed in response to low-temperature stress. The induction of low-DP fructan under low-temperature stress can be due to the synthesis and/or by degradation of available high-DP fructans. Partial degradation of high-DP fructans by FEHs can also increase the low-DP fructan content (Valluru and Van den Ende, 2008). However, induction of low-DP fructans during vacuolar antioxidant mechanisms has also been suggested. A recent *in vitro* study on the scavenging capacity for hydroxyl radicals (·OH) of fructans suggests that fructans might play a role in neutralizing ROS leading to carbon-centered radicals that can be re-utilized for non-enzymatic *de novo* synthesis of fructan oligosaccharides (Peshev et al., 2013). However, the role of fructan in vacuolar antioxidant mechanisms leading to the carbon-centered radicals based *de novo* synthesis of fructan oligosaccharides is to be further investigated.

Low-DP fructan induced during cold acclimation might be involved in protective mechanisms such as membrane stabilization. The effect of fructan DP on liposome stability has been studied using seven defined size classes of fructans from perennial ryegrass and oat, showing that the fructans with a DP4 is more protective than other fractions in liposome fusion (Hincha et al., 2007). Our results show the induction of fructans with a DP4 to the highest levels during cold acclimation. Low-DP fructans might be used for carbon translocation due to their high mobility compared to high-DP fructans. They might also be used as signaling molecules during abiotic stresses (Van den Ende, 2013). Apart from fructan molecules with a specific DP, compositional changes in the mixture of fructan oligosaccharides and polysaccharides might also be important in abiotic stress tolerance.

### Fructan storage in roots as an adaptive trait for plant recovery after freezing

Morphological, physiological, and biochemical changes in the root system during cold acclimation are important determinants of overwintering capacity and recovery of plants. Root system has been suggested as the site of perception of low-temperature stimulus (Goulas et al., 2003). However, not only the signal transduction from root to top, but also from top to root is important for cold adaptation (Ahamed et al., 2012). Rearrangements of cytoskeletal structures in response to low-temperature stress are important for morphological and physiological changes in roots required for the acquisition of freezing tolerance (Kerr and Carter, 1990). Plasma membrane lipid alterations such as compositional changes in fatty acids have also been shown as adaptive traits during cold acclimation (Lee et al., 2005; Sassaki et al., 2013). Exposure of rice roots to low-temperature stress resulted in a gradual increase of root water uptake and induction of genes encoding aquaporins (Ahamed et al., 2012). Aquaporins in root cell membranes are important to maintain the hydraulic conductivity in roots. Accumulation of cryoprotectants, antioxidants, antifreeze proteins, compatible solutes such as WSCs and water soluble proteins, and proteins involved in protective mechanisms in roots indicates numerous metabolic rearrangements in roots during cold acclimation (Zhao and Blumwald, 1998; Espevig et al., 2012; Radkova et al., 2014). Fructan accumulation in roots is also an important metabolic rearrangement in temperate grasses (Chatterton et al., 1989; Prud'homme et al., 1993; Puebla et al., 1997).

“Veyo” originates in the Mediterranean region, and drought-induced summer dormancy has been reported to influence fructan quantity and DP (Volaire et al., 1998; Volaire and Norton, 2006). Accumulation of fructan can be an important strategy for osmoregulation and maintenance of water states in roots when the soil-water availability is limited (Da Silva et al., 2013). Therefore, the presence of more fructans in “Veyo,” compared to “Falster,” at 20°C might be an adaptive trait to drought-induced summer dormancy. “Falster” had increased total fructan content in roots during cold acclimation, and the longer the cold acclimation period continued, the more high-DP fructans were induced. The cold acclimated plants of “Falster” with higher fructan content in roots recovered after freezing faster than “Veyo.” Our results suggest that the lower fructan content in “Veyo” roots, compared to “Falster” roots, could explain the lower adaptability of “Veyo” to freezing. In addition, “Veyo” had a lower capacity to produce high-DP fructans in the roots than in the top. This is supported by the lower level of *Lp1-FFT* expression in the roots than in green tissues (Figure 7C). Apart from the induction of fructan related genes, post-transcriptional mechanisms regulating the function of proteins (Kooiker et al., 2013; Miura and Furumoto, 2013) might also be involved in spatio-temporal dynamics of fructan metabolism during cold acclimation. Further investigation of such mechanisms is important for a better understanding of fructan metabolism.

Hydrolysis of fructan, especially the high-DP fructans in roots might increase osmolality of cell sap rapidly in response to low-temperature stresses. Fructan storage in roots as a carbon sink can be utilized for a rapid growth in early spring to compete with neighboring species as suggested by Van den Ende et al. (2000). This might be also an important adaptive trait of plants for recovery after the destruction of above-ground biomass due to the adverse factors such as freezing, temperature fluctuations, and disease. The ability to store fructan in roots might explain why Pollock and co-workers found only a poor correlation between freezing tolerance and fructan content in two contrasting cultivars of perennial ryegrass as they did not consider the fructan content and composition in roots (Pollock et al., 1988). Overall, our results showed the quantitative and compositional changes in fructan, and the expression patterns of *FTs* and *FEHs* during cold acclimation in two types of perennial ryegrass adapted to different climatic conditions. Our results suggest that accumulation of high-DP fructan in roots as an adaptive trait for plant recovery during cold. The effects of compositional changes of fructan in membrane stability and other protective mechanisms are to be further studied.

### Conflict of interest statement

The authors declare that the research was conducted in the absence of any commercial or financial relationships that could be construed as a potential conflict of interest.
