# Supplementary material for: Fructan metabolism and changes in fructan composition during cold acclimation in perennial ryegrass
Source: Front Plant Sci. 2015 May 12;6:329. doi: 10.3389/fpls.2015.00329 (PMC4428078; doi:10.3389/fpls.2015.00329)
Supplement: Supplementary file 1 [file Presentation1.PDF]

Supplementary Material

Fructan metabolism and changes in fructan composition during cold acclimation in perennial ryegrass

Shamila Weerakoon Abeynayake[1, 2], Thomas Povl Etzerodt[1], Kristina Jonavičienė[3], Stephen Byrne[2], Torben Asp[2], Birte Boelt[1*]

[1] Department of Agroecology, Aarhus University, Forsøgsvej 1, DK-4200 Slagelse, Denmark.

[2] Department of Molecular Biology and Genetics, Aarhus University, Forsøgsvej 1, DK-4200 Slagelse, Denmark.

[3]Laboratory of Genetics and Physiology, Institute of Agriculture, Lithuanian Research Centre for Agriculture and Forestry, Instituto av. 1, LT-58344 Akademija, Kėdainiai distr, Lithuania.

[*]**Correspondence:** Dr. Birte Boelt, Aarhus University, Department of Agroecology – Crop Health, Forsøgsvej 1, 4200 Slagelse, Denmark

Birte.Boelt@agro.au.dk

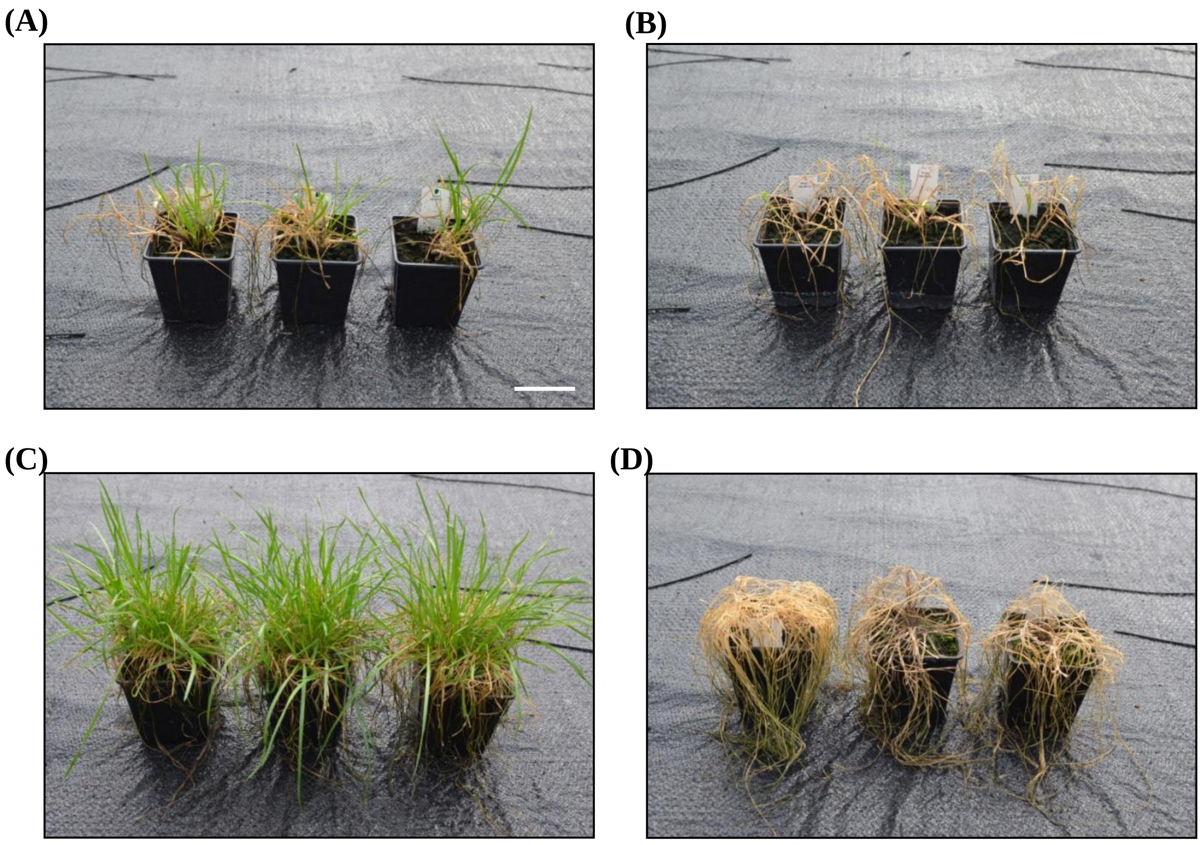

Supplementary Fig. S1. Recovery of cold-acclimated perennial ryegrass (*Lolium perenne* L.) variety ‘Veyo’ and ecotype ‘Falster’ after freezing. (A) Cold-acclimated ‘Veyo’ plants after 7 d of recovery from freezing (–10 °C for 3 h). (B) Non-cold-acclimated ‘Veyo’ plants after 7 d of recovery from freezing. (C) Cold-acclimated ‘Falster’ plants after 7 d of recovery from freezing. (D) Non-cold-acclimated ‘Falster’ plants after 7 d of recovery from freezing. Scale bar, 7 cm.

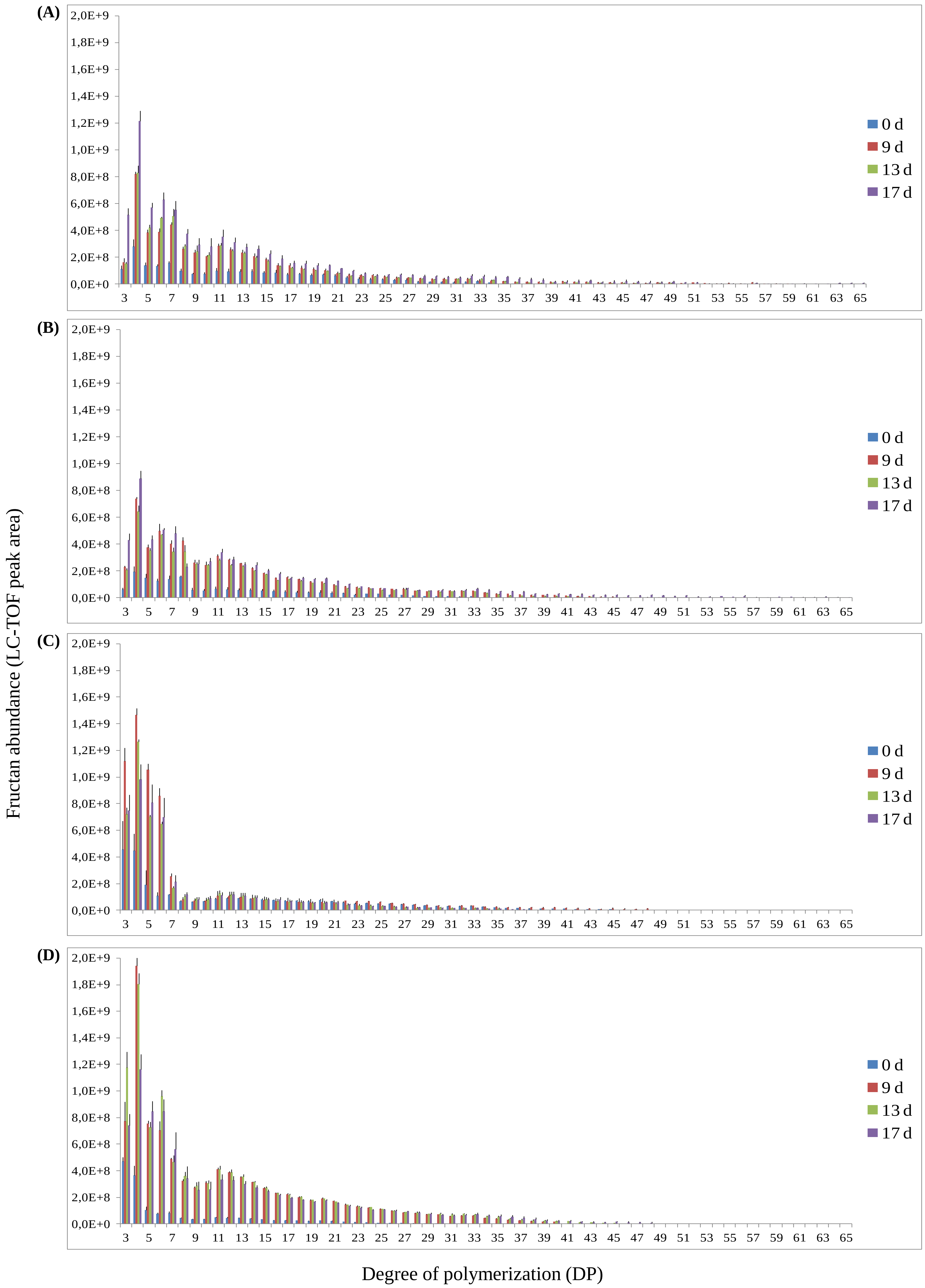

Supplementary Fig. S2. Changes in fructan DP and composition in perennial ryegrass (*Lolium perenne* L.) variety 'Veyo' and ecotype 'Falster' during cold acclimation. Changes in fructan DP and composition in the (A) green tissues (top) of 'Veyo', (B) top of 'Falster', (C) roots of 'Veyo' and (D) roots of 'Falster'. Fructan DP and composition on d 0, 9, 13, and 17 of cold acclimation are shown. Relative quantification of fructan oligosaccharides and polysaccharides was carried out using LC-ESI-TOF MS. Data represent mean ± SE obtained from three replicates of the analysis.
