# Supplementary material for: Fructan metabolism and changes in fructan composition during cold acclimation in perennial ryegrass
Source: Front Plant Sci. 2015 May 12;6:329. doi: 10.3389/fpls.2015.00329 (PMC4428078; doi:10.3389/fpls.2015.00329)
Supplement: Supplementary file 2 [file DataSheet1.PDF]

Supplementary Material

Fructan metabolism and changes in fructan composition during cold acclimation in perennial ryegrass

Shamila Weerakoon Abeynayake[1, 2], Thomas Povl Etzerodt[1], Kristina Jonavičienė[3], Stephen Byrne[2], Torben Asp[2], Birte Boelt[1*]

[1] Department of Agroecology, Aarhus University, Forsøgsvej 1, DK-4200 Slagelse, Denmark.

[2] Department of Molecular Biology and Genetics, Aarhus University, Forsøgsvej 1, DK-4200 Slagelse, Denmark.

[3]Laboratory of Genetics and Physiology, Institute of Agriculture, Lithuanian Research Centre for Agriculture and Forestry, Instituto av. 1, LT-58344 Akademija, Kėdainiai distr, Lithuania.

[*]**Correspondence:** Dr. Birte Boelt, Aarhus University, Department of Agroecology – Crop Health, Forsøgsvej 1, 4200 Slagelse, Denmark

Birte.Boelt@agro.au.dk

Supplementary Table S1. Liquid chromatography TOF-MS characteristics for the major individual fructan species observed in the plant samples. DP corresponding to number of carbohydrate monomers in fructan polymer. Exact mass is the calculated exact for different fructan species in the MassHunter qualitative analysis software. Retention time (tR) of the different major fructan polymers are shown. The observed charges for the different fructan species are marked by grey color in the table. The ratio of exact mass-to-charge was used to extract ion chromatograms (EICs) for fructan polymers in the MassHunter qualitative LCTOF analysis software. Where multiple charges exist for a fructan polymer, EICs are accumulated from species of different charges.

				Charge				
**DP**	**Chemical formula**	**Exact mass [Da]**	**tR [min]**	-1	-2	-3	-4	-5
3	$C_{12}H_{22}O_{11}$	342.1162	2.3					
4	$C_{18}H_{32}O_{16}$	504.1690	2.7					
5	$C_{24}H_{42}O_{21}$	666.2219	3.3					
6	$C_{30}H_{52}O_{26}$	828.2747	4.9					
7	$C_{36}H_{62}O_{31}$	990.3275	7.6					
8	$C_{42}H_{72}O_{36}$	1152.3803	10.1					
9	$C_{48}H_{82}O_{41}$	1314.4332	11.9					
10	$C_{54}H_{92}O_{46}$	1476.4860	13.2					
11	$C_{60}H_{102}O_{51}$	1638.5388	14.2					

12	$C_{66}H_{112}O_{56}$	1800.5916	15.0
13	$C_{72}H_{122}O_{61}$	1962.6444	15.8
14	$C_{78}H_{132}O_{66}$	2124.6973	16.5
15	$C_{84}H_{142}O_{71}$	2286.7501	17.1
16	$C_{90}H_{152}O_{76}$	2448.8029	17.6
17	$C_{96}H_{162}O_{81}$	2610.8557	18.1
18	$C_{102}H_{172}O_{86}$	2772.9086	18.5
19	$C_{108}H_{182}O_{91}$	2934.9614	18.9
20	$C_{114}H_{192}O_{96}$	3097.0142	19.3
21	$C_{120}H_{202}O_{101}$	3259.0670	19.7
22	$C_{126}H_{212}O_{106}$	3421.1199	20.0
23	$C_{132}H_{222}O_{111}$	3583.1727	20.3
24	$C_{138}H_{232}O_{116}$	3745.2255	20.6
25	$C_{144}H_{242}O_{121}$	3907.2783	20.9
26	$C_{150}H_{252}O_{126}$	4069.3312	21.1
27	$C_{156}H_{262}O_{131}$	4231.3840	21.4
28	$C_{162}H_{272}O_{136}$	4393.4368	21.6
29	$C_{168}H_{282}O_{141}$	4555.4896	21.8
30	$C_{174}H_{292}O_{146}$	4717.5424	22.0
31	$C_{180}H_{302}O_{151}$	4879.5953	22.2
32	$C_{186}H_{312}O_{156}$	5041.6481	22.4
33	$C_{192}H_{322}O_{161}$	5203.7009	22.6
34	$C_{198}H_{332}O_{166}$	5365.7537	22.8
35	$C_{204}H_{342}O_{171}$	5527.8066	23.0
36	$C_{210}H_{352}O_{176}$	5689.8594	23.2
37	$C_{216}H_{362}O_{181}$	5851.9122	23.3
38	$C_{222}H_{372}O_{186}$	6013.9650	23.5
39	$C_{228}H_{382}O_{191}$	6176.0179	23.6
40	$C_{234}H_{392}O_{196}$	6338.0707	23.8
41	$C_{240}H_{402}O_{201}$	6500.1235	23.8
42	$C_{246}H_{412}O_{206}$	6662.1763	24.1
43	$C_{252}H_{422}O_{211}$	6824.2291	24.2
44	$C_{258}H_{432}O_{216}$	6986.2820	24.4
45	$C_{264}H_{442}O_{221}$	7148.3348	24.5
46	$C_{270}H_{452}O_{226}$	7310.3876	24.6
47	$C_{276}H_{462}O_{231}$	7472.4404	24.7
48	$C_{282}H_{472}O_{236}$	7634.4933	24.9
49	$C_{288}H_{482}O_{241}$	7796.5461	25.0
50	$C_{294}H_{492}O_{246}$	7958.5989	25.1
51	$C_{300}H_{502}O_{251}$	8120.6517	25.2
52	$C_{306}H_{512}O_{256}$	8282.7046	25.2
53	$C_{312}H_{522}O_{261}$	8444.7574	25.4
54	$C_{318}H_{532}O_{266}$	8606.8102	25.6
55	$C_{324}H_{542}O_{271}$	8768.8630	25.6

56	$C_{330}H_{552}O_{276}$	8930.9159	25.7		
57	$C_{336}H_{562}O_{281}$	9092.9687	25.8		
58	$C_{342}H_{572}O_{286}$	9255.0215	25.9		
59	$C_{348}H_{582}O_{291}$	9417.0743	26.0		
60	$C_{354}H_{592}O_{296}$	9579.1271	26.1		
61	$C_{360}H_{602}O_{301}$	9741.1800	26.2		
62	$C_{366}H_{612}O_{306}$	9903.2328	26.3		
63	$C_{372}H_{622}O_{311}$	10065.2856	26.5		
64	$C_{378}H_{632}O_{316}$	10227.3384	26.5		
65	$C_{384}H_{642}O_{321}$	10389.3913	26.1		
66	$C_{390}H_{652}O_{326}$	10551.4441	26.6		
67	$C_{396}H_{662}O_{331}$	10713.4969	26.7		
68	$C_{402}H_{672}O_{336}$	10875.5497	26.7		
69	$C_{408}H_{682}O_{341}$	11037.6026	27.0		
70	$C_{414}H_{692}O_{346}$	11199.6554	27.9		

Supplementary Table S2. PCR primers used to amplify the template DNA for absolute quantification of gene expression using quantitative RT-PCR. Forward and reverse primers used to amplify the template DNA for the expression analysis of the genes encode sucrose-sucrose 1-fructosyltransferase (Lp1-SST), fructan-fructan 1-fructosyltransferase (Lp1-FFT), fructan-fructan 6G-fructosyltransferase (Lp6G-FFT), fructan exohydrolases (Lp1-FEH and Lp6-FEH), elongation factor 1-alpha (LpEF1a), actin (LpACT11) and eukaryotic initiation factor 4A (LpeIF4a).

Gene	PCR primers (5'-3')	
Lp1-SST	Forward	TTTTCGGTGAGGGTGCTAGT
	Reverse	GTTGTCAGCTGAGGCCATCT
Lp1-FFT	Forward	ACAGCGGAGAGACTCATCGT
	Reverse	GGGGCTTGCTACAACAAGTAA
Lp6G-FFT	Forward	CTGACGGCTCTCTTTGGACT
	Reverse	GAAGTCAGGATGCCTCGTTG
Lp1-FEH	Forward	AGGGTCTGCATAACGTCCAG
	Reverse	CCCTGAATTACATTCACTTGTGC
Lp6-FEH	Forward	GGTCTGCATAACAGCCAGGA
	Reverse	CCCTTATTCACATTCACTTGTGC
LpEF1a	Forward	TGGCTTGCAGTCTCAATAGC
	Reverse	ATCAAGGGCGTGGAGAAGA
LpACT11	Forward	CCCTGCGAGACTAATAATGG
	Reverse	ACTGCCAGCAGATACCGAGT
LpeIF4a	Forward	GGCAGGGATCACACAGTTTC
	Reverse	GTTGATTGCAACACCCTTCC

Supplementary Table S3. Gene-specific primers used for quantitative RT-PCR analysis. Forward and reverse gene specific primers used for the expression analysis of the genes encode sucrose-sucrose 1-fructosyltransferase (Lp1-SST), fructan-fructan 1-fructosyltransferase (Lp1-FFT), fructan-fructan 6G-fructosyltransferase (Lp6G-FFT), fructan exohydrolases (Lp1-FEH and Lp6-

FEH), elongation factor 1-alpha (LpEF1a), actin (LpACT11) and eukaryotic initiation factor 4A (LpeIF4a).

Gene	RT-PCR primers (5’-3’)	
Lp1-SST	Forward	TCCATCGTGCAGAGCTTC
	Reverse	CGTTGTTGAAGAGGTAGACC
Lp1-FFT	Forward	ACCGTCATGGTTAAGGACTC
	Reverse	CGGTCTGATAGCTCATGTG
Lp6G-FFT	Forward	CTGGACCAGAAGGTGTTGG
	Reverse	CACCATCGTGTCATCAGGTG
Lp1-FEH	Forward	GGTCTGCATAACGTCCAGAG
	Reverse	GTGTCGTTGTCGGCAAGTG
Lp6-FEH	Forward	GGTCTGCATAACAGCCAGGA
	Reverse	ACCCTGCCATCGACAAGTG
LpEF1a	Forward	ACTTAGCATACTGGTGTTAGGC
	Reverse	GGTGCCAAGGTTACCAAGG
LpACT11	Forward	TCTGAGTTGCTTGAGGTCAG
	Reverse	CCGAGTGACTAAGACAGAAC
LpeIF4a	Forward	CGTGTGCTCATCACCACTG
	Reverse	CGATGGAGGTAGTTCTCTGG
